# The Promise and Pitfalls of AAV-Mediated Gene Therapy for Duchenne Muscular Dystrophy

**DOI:** 10.3390/cimb47121058

**Published:** 2025-12-17

**Authors:** Elizaveta V. Kurshakova, Olga A. Levchenko, Svetlana A. Smirnikhina, Alexander V. Lavrov

**Affiliations:** Research Centre for Medical Genetics, 115522 Moscow, Russia; kurshakovalv@mail.ru (E.V.K.);

**Keywords:** DMD, AAV, gene therapy, CRISPR/Cas9

## Abstract

Duchenne muscular dystrophy (DMD) is a severe X-linked hereditary disorder caused by pathogenic variants in the *DMD* gene encoding the dystrophin protein. The absence of functional dystrophin leads to destabilization of the dystrophin-associated glycoprotein complex (DAPC), sarcolemmal damage, and progressive degeneration of muscle fibers. Current therapeutic strategies focus on restoring dystrophin expression using genome editing approaches. Adeno-associated virus (AAV) vectors represent the primary delivery platform due to their strong tropism for muscle tissue, low immunogenicity, and ability to achieve long-term transgene expression. However, the limited packaging capacity of AAV (~4.7 kb) necessitates the use of truncated mini- and micro-dystrophin transgenes as well as compact genome editing systems (SaCas9, NmeCas9, Cas12f, TIGR-Tas, and others). Major challenges include immune responses against the viral capsid and transgene products, as well as the inability to perform repeated administrations. Moreover, the duration of expression is limited by the episomal nature of AAV genomes and their loss during muscle fiber regeneration. Despite substantial progress, unresolved issues concerning safety, immunogenicity, and stability of genetic correction remain, defining the key directions for future research in DMD therapy.

## 1. Introduction

Duchenne muscular dystrophy (DMD) is one of the most severe inherited neuromuscular disorders, characterized by relentlessly progressive muscle degeneration and early loss of ambulation. The disease is caused by pathogenic variants in the *DMD* gene encoding dystrophin. DMD is inherited in an X-linked recessive manner, which explains its predominant manifestation in males. The *DMD* gene is the largest known human gene, spanning approximately 2.5 million base pairs and comprising 79 exons [1]. A distinctive feature of *DMD* gene is the presence of seven independent promoters and extensive alternative splicing, which enables the production of multiple dystrophin isoforms (Dp427, Dp140, Dp116, Dp71, and others) expressed in diverse tissues including skeletal and smooth muscle, cardiomyocytes, and cells of the central nervous system [2,3].

**Figure 1:**
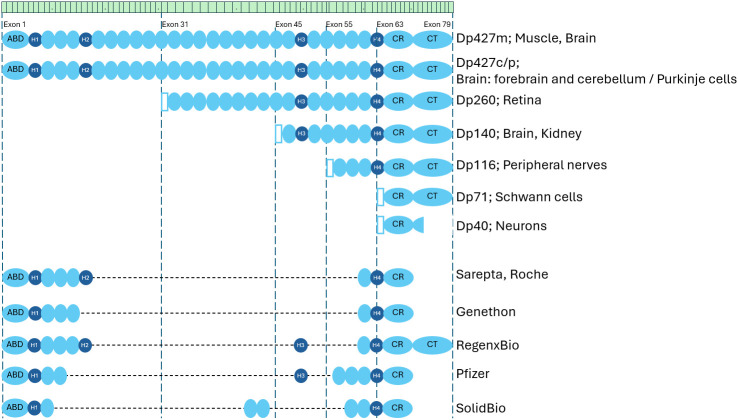
Isoforms of dystrophin and microdystrophins used in clinical trials. ABD—Actin-binding domain; CR—Cysteine-rich domain; CT—C-terminus domain; blue oval—Spectrin-like repeat; dark-blue circle—Hinge; green bar illustrates exons.

The full-length dystrophin isoform (Dp427) is a large cytoskeletal protein with a molecular mass of about 427 kDa, consisting of 3685 amino acid residues and encoded by a mature mRNA of approximately 14 kb [4]. Structurally, dystrophin comprises four highly conserved domains: an N-terminal actin-binding domain, a central rod domain composed of spectrin-like repeats, a cysteine-rich domain, and a C-terminal domain responsible for interactions with components of the dystrophin-associated glycoprotein complex (DAPC) [5] (Figure 1). Dystrophin serves as a key structural element of the DAPC, a multiprotein assembly localized at the sarcolemma of muscle fibers [6]. In addition to dystrophin, the complex includes dystroglycans, sarcoglycans, syntrophins, and dystrobrevins. The correct spatial organization of all DAPC subunits critically depends on the presence of dystrophin, which serves as the central linking element. The DAPC provides mechanical stabilization of the muscle cell membrane during contraction–relaxation cycles by anchoring the intracellular actin cytoskeleton to the extracellular matrix. Thus, the complex plays a fundamental role in maintaining the structural integrity of muscle fibers and in the transmission of contractile force [7].

Pathogenic variants in *DMD* are most commonly represented by large deletions (50–65%) and duplications (10–15%) of exons that induce frameshifts and generate premature stop codons. The resulting transcripts degrade via the nonsense-mediated decay (NMD) pathway, leading to a complete or near-complete absence of functional dystrophin, which disrupts the connection between the cytoskeleton and the extracellular matrix of muscle cells. Loss of dystrophin leads to destabilization of the dystrophin-associated glycoprotein complex (DAPC), resulting in abnormal calcium influx and subsequent excitotoxicity, since the proteins associated with the DAPC include the membrane Ca2^+^-ATPase, calcium-store-regulated channels, and stretch-activated channels [8]. Moreover, in Duchenne muscular dystrophy, neuromuscular transmission is impaired because the threshold required for sarcolemmal depolarization can no longer be reached. This failure to achieve adequate depolarization disrupts signal propagation and ultimately contributes to muscle weakness [9]. Dystrophin also functions as a microtubule-associated protein; therefore, its absence disrupts microtubule stability and organization. The microtubule cytoskeleton normally acts as a mechanotransducer that buffers mechanical strain within muscle fibers. Activation of NADPH oxidase 2 (NOX2), a major source of reactive oxygen species (ROS), occurs through mechanotransduction mediated by the microtubule-associated small GTPase Rac1. During mechanical stretch, Rac1 stimulates NOX2, leading to physiological ROS production. However, when microtubule architecture is disrupted, this pathway becomes excessively activated, resulting in abnormal ROS accumulation and the development of oxidative stress [10]. The dystrophin-associated glycoprotein complex is essential for the proper localization of the neuronal isoform of nitric oxide synthase (nNOS) at the sarcolemma of skeletal muscle fibers, as nNOS binds to the C-terminus of dystrophin. This isoform plays a critical role in glucose metabolism, muscle contractility, and the regulation of muscle blood perfusion. In the absence of dystrophin, nNOS becomes mislocalized at the sarcolemma, destabilized, and subsequently degraded, thereby impairing protective vasodilation and leading to ischemic muscle damage [11,12,13]. Depletion of the regenerative potential of satellite cells further exacerbates muscle degeneration [14]. Collectively, these pathophysiological processes culminate in progressive myofiber necrosis, fibrofatty tissue replacement [15], and systemic manifestations including respiratory insufficiency, dilated cardiomyopathy, and neuropsychiatric complications such as cognitive impairment, autism spectrum disorder, and attention-deficit/hyperactivity disorder [16,17]. In contrast, mutations that preserve the reading frame result in the synthesis of truncated but partially functional dystrophin, causing a milder phenotype known as Becker muscular dystrophy [18,19]. Clinically, DMD affects approximately 1 in 5000 live-born males. Around 65–70% of cases are inherited from carrier mothers, whereas 30–35% arise de novo due to spontaneous mutations [20].

A wide range of therapeutic strategies for Duchenne muscular dystrophy (DMD) are currently under active development, generally classified into two major approaches: the restoration of functional dystrophin expression and the modulation of secondary pathological processes resulting from the absence of the protein. Since existing therapeutic modalities are unable to reverse already established irreversible structural changes, the early initiation of treatment is of critical importance. Despite intensive research efforts, no current therapy fully eliminates the primary cause of the disease; all available interventions remain palliative, aiming to slow disease progression and improve patients’ quality of life [21].

Recent advances have considerably deepened our understanding of the pathogenesis of DMD. Nevertheless, one of the major challenges in developing effective therapeutic approaches remains the efficient delivery of genetic material to striated muscle tissue, which is primarily affected in DMD and constitutes nearly 40% of the total human body mass [22]. Several studies have emphasized that the difficulty of achieving effective gene transfer to skeletal and cardiac muscles arises from both anatomical and structural constraints, including the physical isolation of muscle fibers by fascial layers and the complex organization of the sarcolemma, characterized by numerous invaginations known as T-tubules. Furthermore, the inherent structural and functional heterogeneity of muscular tissues—including skeletal, cardiac, and smooth muscle poses an additional obstacle to the development of universal gene therapy strategies. The fundamental differences in the physiology and gene expression profiles of these muscle types significantly complicate the design of therapeutic approaches capable of achieving uniform transgene expression and functional restoration across all muscle subtypes [23].

Despite these challenges, gene therapy–based approaches offer considerable promise for clinical translation owing to their etiopathogenetic mechanism of action, which targets the primary molecular defects underlying the disease and thus has the potential to prevent or markedly slow disease progression. Among the currently available delivery systems, adeno-associated viral (AAV) vectors are the most extensively studied and widely employed [24]. AAV vectors exhibit several advantageous features, including efficient transduction of postmitotic cells, natural tropism toward muscle tissue, and sustained transgene expression. In addition, they demonstrate superior safety profiles compared with adenoviral and lentiviral vectors, largely due to their low immunogenicity and minimal tendency for genomic integration [25].

## 2. AAV

The adeno-associated virus (AAV) was first identified in 1965 by R. Atchison as a contaminant in adenovirus (Ad) preparations [26]. Although AAV has not been associated with any known human diseases, it has been extensively characterized over the decades following its discovery. Significant progress in the understanding of AAV biology has been facilitated by the relatively simple organization of its genome and the experimental convenience of manipulating it through plasmid-based cloning systems.

AAV lacks the essential genes required for autonomous replication and expression of its own genome. These functions are complemented by coinfection with a helper adenovirus, which provides the RNA genes *E1*, *E2a*, *E4*, and *VA* [27,28]. The AAV genome consists of a single-stranded DNA molecule encoding four major open reading frames (ORFs). The first ORF encodes four Rep proteins (Rep78, Rep68, Rep52, and Rep40) that differ in molecular weight and are involved in viral DNA replication. The second ORF contains the *Cap* gene, responsible for synthesizing three structural capsid proteins—VP1, VP2, and VP3 [29]. The third and fourth ORFs encode the assembly-activating protein (AAP), which facilitates the transport of capsid monomers to the nucleolus assembly site [30], and the recently identified membrane-associated accessory protein (MAAP), whose function remains incompletely understood [31]. The ~4.7 kb viral genome is flanked at both ends by inverted terminal repeats (ITRs) of 145 nucleotides, which serve as self-priming structures during replication and function as packaging signals for Rep-mediated encapsidation of viral DNA [32]. The fundamental principle underlying the design of recombinant adeno-associated viral (rAAV) vectors is that transgene packaging into the capsid becomes possible once all viral coding sequences are removed, retaining only the ITRs, which are indispensable for replication initiation and genome packaging. The viral genome is replaced with an expression cassette comprising a promoter, regulatory elements, and the therapeutic transgene. Importantly, unlike wild-type AAV (wtAAV), recombinant AAV does not integrate site-specifically into the AAVS1 locus of the human chromosome; instead, most rAAV genomes persist as episomal forms within the transduced cells [33]. A major constraint in AAV vector engineering is the limited packaging capacity of the capsid, which can accommodate only approximately 4.7 kb of DNA—roughly the size of the wild-type genome. This imposes strict requirements on the design of recombinant constructs, necessitating careful optimization and minimization of all expression cassette components, including the promoter and coding sequence, to ensure efficient packaging and subsequent transgene expression.

Despite their high transduction efficiency in striated muscle, rAAV vectors exhibit broad tropism for non-skeletal tissues, making the use of tissue-specific promoters essential to restrict transgene expression. Such promoters must ensure strong, long-term, and stable expression in muscle fibers while maintaining minimal activity in other tissue types. Moreover, the limited packaging capacity of AAV requires that these promoters be compact in size. Modern synthetic promoters, such as Spc5-12 and MHCK7, have demonstrated marked advantages over their natural counterparts. These synthetic sequences have been rationally engineered to overcome the packaging limitations of AAV vectors while maintaining high expression efficiency and muscle specificity, as shown in multiple studies [34,35].

The adeno-associated viral (AAV) vector demonstrates exceptional potential as a delivery platform for gene therapy, enabling efficient transfer of both genome-editing system components and truncated gene variants. However, a defining feature that continues to shape the design strategy of AAV-based therapeutics is the limited packaging capacity of the viral capsid, which imposes specific constraints on the construction and optimization of genetic payloads. The delivery of full-length *DMD* cDNA encoding dystrophin using AAV vectors is not feasible due to the large size of the gene [36]. To address this limitation, a shortened yet functional form of the protein was required, with a cDNA size that does not exceed the packaging capacity of the vector. The development of such constructs has proven challenging, primarily because of the complex structural and functional organization of dystrophin. It has been well established that the N-terminal actin-binding domain and the C-terminal domain are both critical for proper protein function and for maintaining the integrity of the dystrophin-associated protein complex (DAPC). Consequently, missense variants located within exons encoding these regions are typically associated with severe disease phenotypes [37]. In addition, the central rod domain—composed of spectrin-like repeats—plays a pivotal role in providing the mechanical stability and elasticity of the protein. Deletions within this region, or the creation of overly truncated constructs, can disrupt the spatial conformation of dystrophin, resulting in functional impairment and accelerated degradation. Nevertheless, clinical observations have reported large in-frame deletions within the central domain that are associated with milder dystrophinopathy phenotypes. For example, a rare case was described in which a deletion encompassing approximately 46% of the coding sequence of the *DMD* gene led to a mild Becker muscular dystrophy phenotype [38].

Analysis of such clinical cases, together with structural studies of dystrophin, has enabled the identification of domains that can be safely removed—individually or in combination—without abolishing protein functionality. This knowledge has laid the foundation for the development of mini- and micro-dystrophins: truncated but functional isoforms capable of recapitulating the key structural and mechanistic roles of full-length dystrophin [39]. These engineered proteins retain only the essential domains, including the N-terminal actin-binding region, four to five spectrin-like repeats, two to three hinge segments, and the C-terminal domain required for integration into the dystroglycan complex.

Preclinical validation of these constructs has been primarily performed using the *mdx* mouse model, which carries a nonsense mutation in the *DMD* gene, resulting in a complete absence of full-length dystrophin expression [40]. Studies employing this model have demonstrated that expression of shortened *DMD* transgenes can almost completely prevent the onset of dystrophic pathology [41,42]. These findings have guided the development of several optimized transgene designs ranging from 3.6 to 4.9 kb in length [39,42,43], compatible with the AAV vector’s packaging limitations while maintaining robust functional efficacy.

At present, the field of gene replacement therapy for Duchenne muscular dystrophy (DMD) is undergoing rapid expansion, as evidenced by the growing number of parallel therapeutic programs being developed by various pharmaceutical and biotechnology companies. In total, 457 clinical studies related to DMD are registered in the ClinicalTrials.gov database, and six studies specifically investigating gene-replacement therapeutic approaches are summarized in the table above (Table 1). An analysis of current clinical developments including SRP-9001 (Sarepta Therapeutics), PF-06939926 (Pfizer), SGT-001/SGT-003 (Solid Biosciences), RGX-202 (Regenxbio), GNT-0004 (Genethon), and INS1201 (Insmed) reveals several key variable parameters that define the individual characteristics of each therapeutic strategy. The principal differences lie in the choice of viral vector serotype (AAVrh74, AAV9, AAV8), the promoter elements employed (MHCK7, MCK, CK8, Spc5-12), and the structural organization of the micro-dystrophin construct itself (ΔR4-R23/CT, Δ3990, μDysSR, cMD1). This variability underscores the multifactorial nature of the optimization process aimed at maximizing the efficacy, tissue specificity, and safety of gene therapy for DMD, where each design component requires meticulous selection and preclinical validation.

The delivery of large nucleases, such as the canonical and most widely used Streptococcus pyogenes Cas9 (spCas9), together with its guide RNA and regulatory elements, is also technically unfeasible due to size constraints. The open reading frame of spCas9 comprises approximately 4.2 kb, nearly exhausting the packaging capacity of the AAV vector [69]. This limitation has driven intensive efforts toward the engineering and identification of compact Cas-family nucleases and their optimized variants compatible with AAV payload restrictions, enabling the development of “all-in-one” gene editing systems.

A naturally occurring ortholog, Staphylococcus aureus Cas9 (saCas9), consists of 1053 amino acids, corresponding to roughly 3.2 kb of coding sequence. This reduced size allows the inclusion of the nuclease, a minimal promoter, and a guide RNA cassette within a single AAV vector. SaCas9 has already demonstrated in vivo efficacy in genome editing experiments, including correction of dystrophin gene mutations in *mdx* mouse models [70]. Similarly sized orthologs such as Neisseria meningitidis Cas9 (NmeCas9, 1082 aa) have been successfully utilized in compact AAV-based systems. Notably, compared with SaCas9, Nme2Cas9 recognizes a simpler PAM sequence (NNNNCC), thereby enabling access to a broader range of genomic loci [71]. The AAV-mediated CRISPR–Nme2Cas9 system has demonstrated efficient editing activity in mammalian cells [72]. An even more compact nuclease, Campylobacter jejuni Cas9 (CjCas9, 984 aa), represents one of the smallest and most packaging-efficient Cas9 orthologs. Owing to its compact size compared to orthologs such as Nme2Cas9, SpCas9, and SaCas9, CjCas9 offers superior payload capacity for viral vectors, including AAV, thereby accommodating additional functional components such as effector domains or homology-directed repair templates. Given its high fidelity in vitro and in vivo, CjCas9 holds strong potential for precise genome manipulation and clinical gene therapy applications [73].

Beyond Cas9 variants, considerable attention has been directed toward Cas12-family nucleases. Certain Cas12a orthologs have been specifically engineered to enhance their activity in mammalian cells while maintaining a relatively small molecular size [74]. The hypercompact Cas12f family (also known as Cas14 or Cas12j) comprises enzymes of only 400–700 amino acids, corresponding to ≤1.5 kb of DNA. Although still in early optimization stages, initial data have demonstrated the feasibility of AAV-mediated delivery and successful genome editing using these miniature systems [75].

Another emerging group, CasΦ (CasPhi) proteins, encoded by bacteriophages, represent ultracompact nucleases with a molecular weight approximately half that of Cas9 or Cas12a (~70 kDa). These enzymes theoretically leave ample genomic space for additional regulatory components within the vector [76].

Most recently, a novel editing system known as TIGR-Tas has been added to the repertoire of AAV-compatible genome editors [77]. Discovered in bacteriophages and parasitic bacteria, the TIGR-Tas complex consists of a dimer of ultra-compact Tas nucleases and a short guide RNA, termed tigRNA (~36 nt). A distinctive feature of TIGR-Tas is its exceptionally high targeting specificity, achieved through a tigRNA sequence complementary to both sense and antisense strands, and the absence of PAM dependence, enabling flexible targeting of virtually any genomic locus. Furthermore, the TasR protein comprises only 331 amino acids, in stark contrast to the 1368 amino acids of SpCas9, making TIGR-Tas one of the most compact gene editing systems identified to date and ideally suited for AAV packaging, with sufficient remaining space to include promoter elements, polyadenylation signals, and guide RNA cassettes.

Contemporary advances in the development of compact Cas systems are thus paving the way for next-generation AAV vectors capable of co-delivery of both nuclease and guide RNA within a single construct (“all-in-one” vectors). In the context of Duchenne muscular dystrophy, such innovations hold the promise of achieving safer and more effective restoration of dystrophin expression through optimized and clinically viable delivery strategies. The characteristics of the previously discussed nucleases are summarized in Table 2.

Despite the evident advantages of AAV vectors as platforms for delivering truncated gene forms and genome editing components, a major challenge in clinical application remains the development of immune responses. Long-term efficacy and safety of gene therapy are substantially limited by immunogenicity, which may arise from both pre-existing immunity and treatment-induced immune activation. This immune response may target not only the AAV capsid but also the transgene product and, in the case of genome editing systems, the bacterial domains of Cas nucleases. Therefore, comprehensive immunogenicity assessment represents a critical step in the rational design of safe and effective gene therapies.

## 3. Immune Response

Adeno-associated viruses (AAVs) are small, non-enveloped viral particles that are considered non-pathogenic, a property that underlies their dependence on a helper virus (such as adenovirus) for productive replication under natural conditions [78,79]. Recombinant AAV-based vectors, in which native viral genes are replaced by a therapeutic transgene cassette, are characterized by low immunogenicity and demonstrate a generally favorable tolerability profile [79]. Data accumulated from ongoing clinical trials of AAV-mediated gene therapies have yielded encouraging results across multiple disease contexts [80,81,82]. However, years of clinical experience have revealed several persistent and, in some cases, unanticipated adverse events associated with AAV-mediated gene transfer. The most significant challenges include immune-mediated toxicity and the need to overcome neutralizing antibody responses directed against AAV capsid proteins.

A major factor limiting the broad application of AAV-based gene therapy is the requirement for high vector doses, which are typically calculated on a per kilogram basis. In Europe, the mean age of Duchenne muscular dystrophy (DMD) diagnosis is approximately 4.5 years, while in Russia (where the average age at diagnosis is 6.5 years [83]), patients tend to begin therapy substantially later. By the age of 4–5 years, a child already possesses a relatively high body mass, necessitating administration of large absolute quantities of viral particles. In older patients (aged 6–7 years and above), the required vector dose increases even further, proportionally elevating the antigenic load and the associated risk of severe immune-mediated complications, including immune responses targeting both the AAV capsid and the transgene product. In clinical studies, administered doses have ranged from 5 × 10^13^ to 2 × 10^14^ vector genomes per kilogram of body weight [82,84]. Use of vectors within this range has been associated with several dose-dependent toxic effects, primarily resulting from innate immune responses to the AAV vector. These effects include hepatotoxicity (often mediated by effector T-cell responses to viral capsid proteins or the transgene product), thrombocytopenia, erythrocyte hemolysis, microvascular injury such as thrombotic microangiopathy (TMA), and cardiotoxicity [85]. Such adverse events have been documented in clinical trials of gene therapies for Duchenne muscular dystrophy.

The accumulated experience from AAV-based therapies provides valuable insight into adverse effects not only in DMD but also in other genetic disorders. For instance, early clinical trials of AAV-mediated gene therapy for hemophilia B reported a rapid decline in the initially achieved expression of the delivered coagulation factor IX, accompanied by an elevation of hepatic enzyme levels []. Subsequent investigations identified this phenomenon as a result of capsid-specific T-cell activation, leading to the elimination of transduced hepatocytes and consequently the loss of therapeutic efficacy [86,87]. Current pathogenetic models suggest that clearance of transduced cells is mediated by a cytotoxic immune response directed against AAV capsid-derived antigens [88]. Although the target cells—such as hepatocytes—are not professional antigen-presenting cells, degradation of capsid proteins within these cells can lead to the presentation of viral peptides in complex with MHC class I molecules. Simultaneously, capsid-derived epitopes can be processed and presented on MHC class II molecules by professional antigen-presenting cells such as dendritic cells, initiating the activation of capsid-specific CD4^+^ T-helper and CD8^+^ cytotoxic T-lymphocytes [88]. Activated CD8^+^ T-cells subsequently recognize and lyse transduced cells presenting capsid epitopes on MHC I, resulting in loss of genetic correction and diminished therapeutic effect. To mitigate this issue, immunosuppressive therapy has been employed: corticosteroids have demonstrated efficacy in suppressing immune responses in most reported cases [88]. Consequently, nearly all current clinical protocols incorporate prophylactic or reactive immunosuppression to prevent the loss of corrected cells. Nevertheless, corticosteroid use is itself associated with adverse effects [89,90]. Importantly, the risk of T-cell-mediated immune responses is dose-dependent and remains a major challenge for achieving long-term stable gene correction, particularly when high vector doses are required.

The AAV capsid is widely distributed in the human population [91]. The high prevalence of natural AAV infection, particularly in children, results in the development of pre-existing humoral and cell-mediated immunity in a substantial proportion of individuals [92]. The presence of neutralizing antibodies (NAbs) can inhibit the transduction process by blocking vector entry into target cells, thereby substantially reducing therapeutic efficacy [93]. The frequency of pre-existing NAbs varies markedly depending on the AAV serotype. The highest seroprevalence rates, exceeding 50%, are typically observed for AAV2, with antibody titers often reaching levels that preclude its therapeutic use. Other serotypes, such as AAV8 and AAV9, demonstrate lower seroprevalence; however, cross-reactivity among serotypes remains a significant limiting factor [94]. Consequently, pre-screening for anti-AAV antibodies specific to the intended vector serotype is a mandatory prerequisite before AAV-based gene therapy administration. Following vector delivery, patients typically develop a robust and long-lasting humoral immune response with high-titer NAbs [95], which creates an absolute contraindication for vector re-administration [96]. This limitation poses a major obstacle for patients whose initial treatment proves suboptimal or who later experience elimination of corrected cells due to immune-mediated mechanisms. At present, no safe and effective protocols exist for repeat AAV vector administration in such individuals.

Following AAV-based therapy, post-treatment monitoring is essential to detect potential adverse events. AAV vectors tend to accumulate in the liver, creating a risk of dose-dependent hepatotoxicity. Reported clinical observations include transient renal impairment and temporary elevations in liver enzyme levels [97]. Furthermore, vector administration induces de novo production of specific antibodies, rendering re-dosing infeasible due to the high likelihood of vector neutralization upon re-exposure [98].

The analysis of adverse outcomes, including fatal events, in clinical studies employing adeno-associated virus (AAV) vectors allows us to conclude that a causal relationship exists between administration of very high vector doses and the development of a severe systemic immune response. For example, one report described the death of a patient approximately one month after intravenous administration of a high-dose rAAV9 vector, with post-mortem examination revealing multiorgan failure, severe hypoxic–ischemic neurologic injury, and diffuse alveolar damage [99,100]. These tragic events underscore the critical risks associated with dose-dependent immunogenicity of the AAV vector, and highlight the urgent need for development of safer dose profiles, utilization of plasma apheresis, immunomodulatory therapies, or selection of alternative viral serotypes. Current research is actively exploring the efficacy of these mitigations [97,101].

Likewise, a serious unresolved problem remains the immune response directed against the delivered transgene. The introduction of any dystrophin variant (including micro- or mini-dystrophins) into patients whose full-length dystrophin has been absent since birth may provoke both humoral and cellular immune responses. In clinical gene-therapy trials, serious adverse events have been reported: muscle weakness, respiratory insufficiency, myocarditis, myositis, muscle swelling with T-cell infiltration. The duration of these reactions correlated with the period of transgene expression, which supports the hypothesis of immune recognition of dystrophin. For example, immuno-assay and epitope-mapping studies demonstrated immunologic reactivity to the peptide region encoded by exons 8–11 in patients with deletions spanning exons 8–21 [102]. Similar results were found in a patient with deletion of exons 3–17 [103]. In the clinical trials of the gene-therapy product Elevidys (delandistrogene moxeparvovec), a correlation was established between patients harboring deletions of exons 8–9 of the DMD gene and the development of immune-mediated reactions. The clinical picture included immune-mediated myositis: muscle weakness, dysphagia, respiratory disturbances (cough, dyspnoea), fever, fatigue and weight loss. In pediatric cohorts, the onset of the reaction was particularly acute. On the basis of these data, therapy is contraindicated in patients with deletions of exons 8–9, as well as deletions encompassing exons 1–17 and/or 59–71, owing to a comparable risk of developing severe immune-mediated reaction [104]. Thus, when considering gene-replacement therapy for DMD, one may infer that only patients with deletions limited to a certain exon interval (e.g., 18–58)—which corresponds to a little over one-half of the gene length—may be appropriate candidates, while those with other exon deletions fall into a high-risk group for immune complications following restored dystrophin expression. It is important to emphasize that not only gene-replacement therapy, but also genome-editing approaches aimed at restoring the reading frame will result in renewed expression of potentially immunogenic dystrophin domains. In this respect the possibility arises of designing micro-dystrophin variants with a rational design that restores function without generating antigenic epitopes—effectively constituting a novel protein.

Key components of genome-editing systems, in particular bacterial nucleases, are likewise recognized by the human immune system as foreign antigens. This is primarily due to the fact that bacteria which are the natural source of these enzymes (e.g., *Staphylococcus aureus*, *Streptococcus pyogenes*) are widely encountered in the human population. Immuno-enzymatic investigations confirm the existence of pre-existing humoral and cell-mediated immunity to SaCas9 and SpCas9 in a considerable fraction (over 80%) of healthy individuals [105]. For overcoming this limitation, strategies are being developed which involve modification of immunodominant epitopes within the Cas9 protein structure. Such modifications allow a substantial reduction in immunogenicity of the nucleases without compromising their catalytic activity or specificity [106].

## 4. Duration of Expression

The question of vector persistence, sustained transgene expression, and, consequently, the long-term correction of pathological phenotypes remains one of the central unresolved challenges in the field of gene therapy. Recombinant adeno-associated virus (AAV) vectors, being non-integrating by nature, persist within the nucleus predominantly in episomal form rather than being incorporated into the host genome [79]. Because AAV vectors lack an intrinsic mechanism of replication, their copy number per cell progressively declines over time in proliferating cell populations due to mitotic dilution. This limitation is of particular importance in pediatric applications, where ongoing growth and development are accompanied by high rates of cellular proliferation in target tissues. Consequently, it becomes difficult to assert that AAV-mediated gene transfer can ensure sustained clinical benefit in diseases that require correction in actively dividing cell populations, especially considering the practical impossibility of repeated systemic vector administration due to the development of neutralizing antibodies.

Cardiomyocytes, as terminally differentiated cells with extremely low proliferative activity, represent an ideal target for achieving stable and durable transgene expression. In contrast, the situation in skeletal muscle is substantially more complex, particularly in the pathological context of Duchenne muscular dystrophy (DMD). Physiological muscle regeneration occurs through the fusion of satellite cells (myosatellites) with damaged myofibers [107], while the formation of new fibers depends on proliferation and differentiation of myogenic precursor cells, efficient transduction of which may be limited [108]. Based on these observations, it has been hypothesized that each cycle of muscle regeneration may result in the progressive dilution of myoblasts harboring the therapeutic transgene, as they fuse with non-transduced satellite cells. This process would gradually lead to loss of episomal AAV DNA encoding micro-dystrophin, and consequently, to a decline in dystrophin expression over time, ultimately allowing disease progression to resume.

Nevertheless, several studies have demonstrated that AAV vectors are capable of sustaining long-term transgene expression in non-dividing or slowly dividing tissues. In the context of micro-dystrophin gene replacement therapy, this persistence may provide a prolonged but not lifelong therapeutic effect, given the gradual attrition of episomes in regenerating skeletal muscle. However, the same property poses a significant safety concern when AAV vectors are employed to deliver components of genome-editing systems, such as CRISPR-associated nucleases. Prolonged expression of these highly active enzymatic proteins within the cell increases the risk of off-target genomic modifications, which may result in genomic instability, oncogene activation, or inactivation of tumor suppressor genes, representing a substantial safety hazard.

Thus, the fundamental mechanism of AAV-mediated transgene expression embodies a critical paradox: while its limited stability may undermine the long-term efficacy of gene replacement therapies, its excessive duration may render genome-editing applications potentially hazardous due to sustained nuclease activity. Addressing this dichotomy remains a pivotal objective in optimizing the balance between therapeutic durability and genomic safety in AAV-based interventions.

## 5. Conclusions

Gene therapy using adeno-associated virus (AAV) vectors represents a transformative approach for treating Duchenne muscular dystrophy (DMD), moving beyond symptomatic management to address the genetic root of the disease. The unique biological properties of AAVs, including their low pathogenicity, ability to transduce non-dividing cells, long-term persistence as episomes, and the availability of various serotypes with tropism for muscle tissue, make them the leading platform for in vivo gene delivery. Significant progress has been made in both gene replacement therapy and gene editing therapy.

However, the clinical application of AAV-based therapies faces several critical challenges that limit their safety and efficacy. The most significant hurdle is the host immune response, which can be directed against the AAV capsid or the newly expressed transgene product. Pre-existing neutralizing antibodies can block transduction, while capsid-specific T-cell responses can lead to the elimination of transduced cells, resulting in loss of therapeutic effect. Furthermore, the high vector doses required to treat the extensive muscle mass in DMD patients are associated with serious adverse events, including hepatotoxicity and thrombotic microangiopathy. Finally, the episomal nature of AAV vectors leads to a gradual decline of transgene expression in regenerating skeletal muscle, raising questions about the long-term durability of the treatment, while persistent expression of gene-editing machinery carries potential risks associated with off-target effects. Possible strategies to mitigate the immune response to AAV include plasmapheresis and the use of immunomodulatory agents.

Future efforts must focus on developing next-generation AAV technologies. This includes engineering novel capsids with enhanced muscle tropism and reduced immunogenicity, optimizing immunosuppressive regimens, and devising strategies for re-dosing. Parallel exploration of non-viral delivery platforms, such as lipid nanoparticles (LNPs) for mRNA and CRISPR components or cell-derived nanovesicles (CDNs), is crucial to overcome the inherent limitations of viral vectors. These alternative systems offer potential advantages, including reduced immunogenicity, high packaging capacity, and the possibility of repeated administration [109]. Ultimately, a diversified toolkit of delivery vectors will be key to creating safe and effective personalized therapies for all DMD patients.

In conclusion, while AAV-based gene therapy for DMD has demonstrated immense promise and is already a clinical reality, overcoming the challenges of immunogenicity, delivery efficiency, and long-term safety is essential to fully realize its potential as a durable and effective treatment for all patients. It is also essential to consider the genetic heterogeneity of patients. Previous clinical data demonstrate that individuals harboring certain large deletions (for example, those spanning exons 1–17 or 59–79) exhibit an increased risk of immune reactions to microdystrophin, necessitating careful consideration during patient selection, informed consent, and risk-benefit assessment. Therefore, ensuring treatment accessibility for all patients requires a personalized approach designed to minimize immunogenicity and brings ethical questions. This entails developing fair frameworks for prioritizing therapies for different exon deletions and establishing robust, long-term safety surveillance to balance hope with realistic expectations for families. Taken together, future progress in AAV-based therapy for DMD will depend on the integration of engineering, clinical, and ethical strategies aimed at achieving safer, more immunologically tolerable, and durable therapeutic outcomes.

## Figures and Tables

**Table 1:** Update in the field of DMD gene replacement therapy.

	Sarepta Ther.	Pfizer	Solid Bio.	Regenxbio	Genethon	Insmed
Gene therapy	SPR-9001 (Elevidys)	PF-06939926	SGT-001, SGT-003	RGX-202	GNT-0004	INS1201
Clinical trials	NCT05096221 EMBARK [44]	NCT04281485 NCT05429372 [45,46]	NCT06138639 NCT03368742 [47,48]	NCT05693142 NCT05683379 [49,50]	GNT-016-MDYF [51]	NCT06817382 [52]
Serotype	rAAVrh74	AAV9	AAV9	AAV8	AAV8	AAV9 intrathecal
Promoter	MHCK7	hCK/MCK	CK8	Spc5-12	Spc5-12	Not disclosed
Microdystrophin structure	∆R4-R23∆CT (4,7 kb DNA) [53]	Δ3990 [54]	μDys5R [55]	µDysCT48 µDysCT194 [56]	hMD1 [57]	pDys [58]
Current status	Completed; A multicenter clinical trial, ENVISION (Study 303, NCT05881408), to evaluate delandistrogene moxeparvovec (SRP-9001, Elevidys) in both ambulatory and non-ambulatory DMD patients [59]	Development discontinued, but patients continue to be monitored [60].	Active, not recruiting. The first dose/primary cohorts were announced and the first patients were dosed in 2024-2025 [48].	NCT05693142 the pivotal Phase I/II/III trial is recruiting >1 y.o. patients. NCT05683379 observational screening study to evaluate the prevalence of AAV8 antibodies in patients up to 12 y.o. [61].	Active, Genethon is approved to begin pivotal Phase 3 clinical trials in France and the UK [57]	Active (phase 1/ASCEND), recruitment/primary dosing was planned/started; there were no public announcements about the program-wide stop/termination [62].
Treatment-related death	Three deaths; FDA Requests Sarepta Therapeutics Suspend Distribution of Elevidys [63,64,65]	At least two documented deaths in research cohorts, serious adverse events led to suspensions and revisions of protocols [66,67].	No documented deaths. Severe adverse events [68].	There are no deaths directly related to the introduction of RGX-202 in open press sources.	There are no deaths directly related to the introduction of GNT-0004 in open press sources.	There are no deaths directly related to the introduction of INS1201 in open press sources.

**Table 2:** Comparative Characteristics of Cas Nucleases for AAV-Mediated Delivery.

Protein (Orthologue/System)	Size (Amino Acids)	Coding Sequence, kb	All-in-One AAV Capability	Applicability
SpCas9 (Streptococcus pyogenes)	1368	~4.1	-	Classic Cas9, widely studied; applicable through separation into two vectors
SaCas9 (Staphylococcus aureus)	1053	~3.2	+	Successfully delivered using single-AAV
NmeCas9 (Neisseria meningitidis)	1082	~3.25	+	Compact orthologue
CjCas9 (Campylobacter jejuni)	984	~2.95	+	One of the smallest natural Cas9
Cas12a (Cpf1)	~1200	~3.6	- (optimization of the promoter and cassette is required)	
Cas12f (Cas14/12j)	400–500	1.2–1.5	+	Hypercompact; successfully active in human cells after engineering optimization
CasΦ (Phage Cas)	~700	~2.1	+	
Mini-Cas9	700–1000	2.1–3.0	+	
TasR (TIGR-Tas)	331	~1.0	++	New system, ultra-compact protein; DNA cutting in human cells proved

## Data Availability

No new data were created or analyzed in this study.
